# Hydra as a model organism to decipher the toxic effects of copper oxide nanorod: Eco-toxicogenomics approach

**DOI:** 10.1038/srep29663

**Published:** 2016-07-15

**Authors:** Anbazhagan Murugadas, Mohammed Zeeshan, Kaliannan Thamaraiselvi, Surendra Ghaskadbi, Mohammad Abdulkader Akbarsha

**Affiliations:** 1 Department of Environmental Biotechnology, Bharathidasan University, Tiruchirappalli 620024, India; 2 Mahatma Gandhi - Doerenkamp Center for Alternatives to Use of Animals in Life Science Education, Bharathidasan University, Tiruchirappalli 620024, India; 3 Developmental Biology Group, MACS-Agharkar Research Institute, Pune 411004, India; 4 Department of Food Science and Nutrition, College of Food and Agriculture, King Saud University, Riyadh 11451, Kingdom of Saudi Arabia

## Abstract

Nanotechnology has emerged as a powerful field of applied research. However, the potential toxicity of nano-materials is a cause of concern. A thorough toxicological investigation is required before a nanomaterial is evaluated for application of any kind. In this context, there is concerted effort to find appropriate test systems to assess the toxicity of nanomaterials. Toxicity of a nanomaterial greatly depends on its physicochemical properties and the biological system with which it interacts. The present research was carried out with a view to generate data on eco-toxicological impacts of copper oxide nanorod (CuO NR) in *Hydra magnipapillata* 105 at organismal, cellular and molecular levels. Exposure of hydra to CuO NR resulted in severe morphological alterations in a concentration- as well as duration-dependent manner. Impairment of feeding, population growth, and regeneration was also observed. *In vivo* and *in vitro* analyses revealed induction of oxidative stress, genotoxicity, and molecular machinery of apoptotic cell death, accompanied by disruption of cell cycle progression. Taken together, CuO nanorod is potentially toxic to the biological systems. Also, hydra offers potential to be used as a convenient model organism for aquatic ecotoxicological risk assessment of nanomaterials.

Among the various 3d transition metals and their oxides, copper oxide (CuO) is a unique monoxide compound which is a p-type semiconductor and a high temperature superconductor, with a narrow band gap of 1.2 eV^1^,^2^. It is used in batteries, magnetic storage media, and gas sensors^3^, as biocide^4^, and as antimicrobial and antifouling agent in paints^5^. Hence, considerable attention has been focused towards synthesis and fabrication of various forms of nano CuO^2^. In the recent past among the various forms of CuO nanomaterials CuO nanorod (CuO NR) has gained importance in view of its large surface area to volume ratio and excellent absorption in the near infra-red region, which makes it an ideal candidate for gas sensing applications^6^,^7^. It has also been found to be the best for near-infra red diffuse reflector and, hence, finds application as NIR obscurant^2^. It has further been shown to be useful as a catalytic agent in ammonia perchlorate decomposition^8^. All these and other applications of CuO NR can lead to large volumes of production which would be a cause of concern in terms of environmental and human health.

Toxicity evaluation of nano-materials has not yet been standardized in view of their varied physicochemical properties such as size, shape, structure, dissolution, agglomeration, surface coating and charge^9^,^10^,^11^. Extensive data on toxicity of nanomaterials have been generated by adopting *in vitro* techniques which represent a very good model system for assessment of human health hazard^11^. Oxidative stress is the major cause of nano CuO-induced toxicity. However, until now there has been no report on the toxicity of CuO NR. It is speculated that nanorod would exhibit greater toxicity in view of its large surface area to-volume ratio which would enhance its interaction with biomolecules resulting in induction of oxidative stress that would lead to cell death^12^. The US National Research Council of the National Academy Sciences envisaged adoption of *in vitro* techniques to understand the toxicity pathways without relying on the laboratory animal models^13^. The common conclusion drawn from various *in vitro* studies testified that CuO nanoparticles induce cytotoxicity through DNA damage and oxidative stress leading to apoptotic cell death^14^,^15^,^16^.

Ecotoxicological risk assessment of CuO nanomaterials mainly focuses on aquatic organisms^10^. In view of the various industrial and domestic applications, storage and/or transportation, nanomaterials can potentially find their way into the aquatic environments which, therefore, need special attention^17^. Nanomaterials, on arrival at the aquatic environments, enter into the biota directly as well as indirectly through bio-accumulation and the food chain and become a serious threat to the aquatic biota^18^. From the aquatic habitats they can access the terrestrial animals and the humans through food chain and inflict toxicity^19^. The European Union (EU) REACH (Registration, Evaluation, Authorization and Restriction of Chemicals) legislation strongly suggests exploitation of aquatic model organisms to evaluate the impacts of chemicals that are sold in the EU markets^20^. Although several studies have been conducted on aquatic organisms such as daphnia, zebrafish and artemia for assessing the toxicity of CuO nanoparticles^21^,^22^,^23^, the toxicity of CuO nanorod (NR) has not yet been evaluated.

Therefore, for the present study we chose *Hydra magnipapillata* 105, a simple freshwater organism belonging to phylum Cnidaria, as the model to evaluate the detrimental effects of CuO NR. Hydra reproduces fast, is easy to culture in the lab and is sensitive to environmental contamination. Since its first description, hydra has been used as a model organism for various aspects of biology such as pattern formation, regeneration, developmental programming, symbiosis, innate immunity^24^ and toxicity testing^25^. Hydra consists of a cylindrical tubular body column, apical hypostome surrounded by tentacles that contain special type of cells called nematocysts, which enable to catch the prey, and a basal disc with which it attaches to the substratum^26^. Hydra is a diploblastic animal with an outer ectoderm and an inner endoderm separated by a non-cellular mesoglea^27^. The ectoderm serves as a protective layer, among other functions, whereas the endoderm lines the entire gastric cavity where digestion of food takes place^28^. All the cells in hydra are in contact with the aqueous environment which facilitates permeation of toxic substances into the animal^29^,^30^. Earlier studies which used hydra as the model in toxicological evaluations revealed severe morphological changes, teratogenicity, delayed growth, alterations of gene expression and induction of apoptosis^25^,^31^,^32^,^33^. The entire genome of hydra having been sequenced, the recent advances in molecular biological techniques enabled us to decipher the mechanisms underlying CuO NR toxicity in hydra from morphopysiological, cellular and molecular perspectives.

## Results

### Characterization of CuO NR

**Figure 1:**
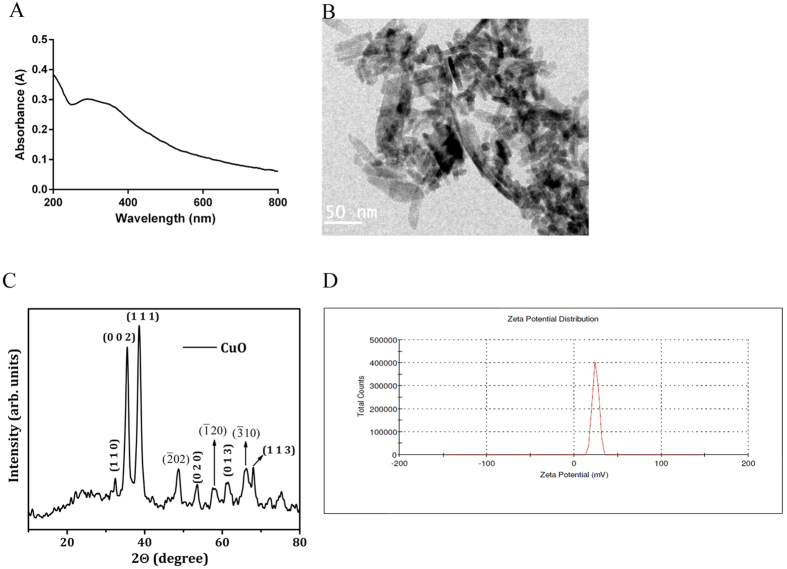
Physico-chemical characterization of CuO NR. (**A**) UV–Visible spectra of the CuO NR dispersed in water; (**B**) TEM image of the CuO NR. (**C**) XRD pattern of the CuO NR; (**D**) Zeta potential of the CuO NR.

The synthesized CuO NR was dispersed in water and the suspension was scanned with the wavelength range of 200 to 900 nm in an UV-Vis Spectrophotometer, which showed a broad absorption peak at 296 nm (Fig. 1A). TEM analysis revealed that the nanoparticles were well dispersed rod-shaped particles measuring a length of 57.69 ± 19.64 nm and breadth of 7.76 ± 1.47 nm. They had smooth surface and blunt ends (Fig. 1B).

The nanorods were further investigated using powder XRD (Fig. 1C). The reflection peaks were identical to the single-phase CuO with a monoclinic structure, and the diffraction data corresponded to the JCPDS card of CuO (JCPDS 80-1268). No peaks of impurity were observed in the XRD pattern. In order to find stability of the CuO NR the zeta potential was measured, and the value recorded was 22 mV (Fig. 1D) which indicated that the nanorods were unstable.

### Determination of median lethal concentration (LC_50_) and uptake of CuO NR

**Figure 2:**
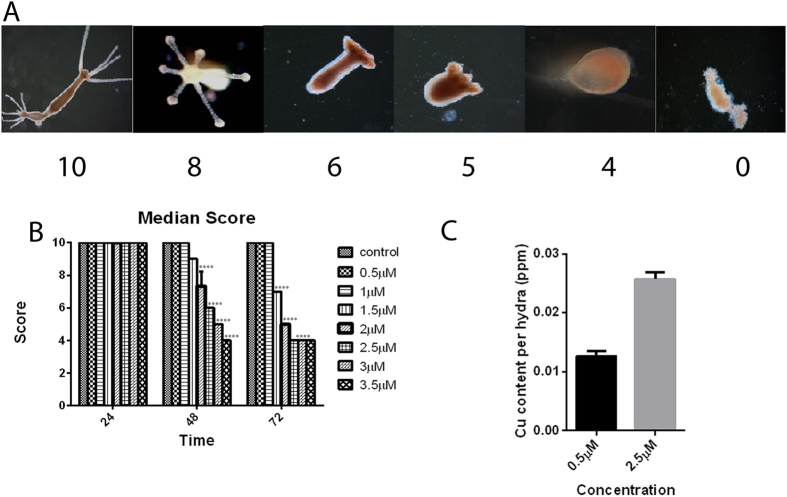
Impact of CuO NR on morphology and viability of hydra. (**A**) Representation of Wilby’s scale of toxicity testing following CuO NR exposure. The complete description of the structural changes associated with the numerical score is provided in the Table S1. (**B**) Median score of polyps exposed to increasing concentrations of CuO NR. Median score was calculated for each concentration and time point on groups of 25 animals. Significance between the control and the treatment groups were performed by adopting two way ANOVA with Dunnet’s multiple comparison test (****p < 0.001)). (**C**) The intracellular Cu content was evaluated by elemental analysis. Polyps were exposed to CuO NR for 24 h. The Cu content is represented in ppm.

CuO NR exposure induced dose- and time-dependent toxicity to hydra, as revealed in the morphological changes measured in the Wilby’s scale^34^ (Fig. 2A). Figure 2B and Supplementary Table S1 reveal that alterations in the median score would enable determination of LC_50_. The LC_50_ (Table 1) was calculated based on time and concentration of CuO NR. LC_50_ determination enabled us to fix the sub-lethal doses to assess the chronic effect of CuO NR. The Cu^2+^ content per hydra after 24 h incubation was dose-dependent (Fig. 2C).

### Alteration of feeding behavior and disruption of battery cell complexes (BCCs)

**Figure 3:**
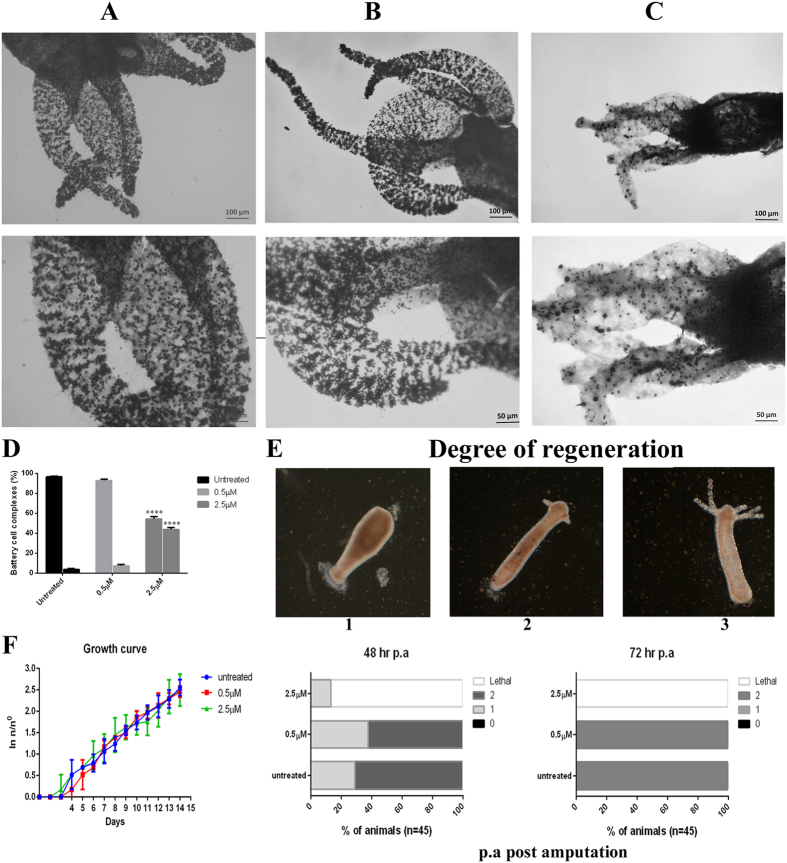
Disruption of battery cell complex, regeneration and reproduction capability in hydra following CuO NR exposure. (**A**) Distribution of battery cell complexes in normal hydra. (**B**) Hydra exposed to 0.5 μM concentration of CuO NR. (**C**) Hydra exposed to 2.5 μM concentration of CuO NR. Upper panel, lower magnification; lower panel, higher magnification. (**D**) Quantitative analysis of disrupted array of battery cell complexes. Bars represent the comparison of percentages of collapsed battery cell complexes between normal and CuO NR exposed hydras following 24 h exposure. Samples of 100 battery cell complexes were accounted for this study. Data are expressed as mean ± SD of three independent experiments. Multiple comparison of significance was performed by adopting two way ANOVA with Dunnet’s multiple comparison test (****p < 0.001). (**E**) Effect of CuO NR on regeneration. Groups of 15 animals were bisected just below hypostome and allowed to regenerate (scheme at the top panel) in the presence of 0.5 μM or 2.5 μM concentration of CuO NR, or in normal medium. At 48 h and 72 h post-amputation animals were examined for viability and regeneration stage: stage 0 indicates complete inhibition of regeneration; stage 1 indicates the presence of tentacle buds; stage 2 indicates new emerging tentacles. Animals exposed to 2.5 μM concentration of CuO NR were significantly impaired in regeneration. Chi-square determined was equal to 273.4 (p < 0.0001). (**F**) Impact of CuO NR on reproduction. Growth test was performed with a population of four adult hydras, either untreated or exposed for 24 h with 0.5 or 2.5 μM concentrations of CuO NR, washed and monitored every day for bud detachment.

Feeding behavior in hydra is a multi-step process that begins with capture of prey followed by opening of the mouth and then insertion of prey into the mouth (Media file, M1). It has been reported that reduced glutathione (GSH) can mimic this multi-step process^35^,^36^. Therefore, we exposed hydra to GSH added to the medium, as a positive control, and found writhing of tentacles followed by their bending towards the mouth (M2). Hydra exposed to 2.5 μM CuO NR for 24 h was challenged with *Artemia* nauplii or GSH. In the presence of live nauplii in the vicinity the polyps were unable to catch the prey (M3) and at the same time GSH response was not inhibited as revealed in tentacle writhing (M4). These results revealed hydra’s inability to catch the prey, which may be due to insufficient nematocyst discharge to induce opening of the mouth. Since battery cells of hydra tentacles play a crucial rule in catching the prey^37^ we assessed if CuO NR exposure produces any impact on the morphology and distribution of BCCs by performing toluidine blue staining followed by observation in a bright field microscope. In a normal polyp an array of BCCs was recorded in the tentacles as seen in Fig. 3A. Exposure to 0.5 μM CuO NR for 24 h did not produce any adverse effect on BCCs (Fig. 3B) whereas 24 h exposure to 2.5 μM CuO NR disrupted the BCCs (Fig. 3C,D).

### Impact of CuO NR on head regeneration and growth capabilities of hydra

Hydra is known for its remarkable ability to regenerate the amputated body region. It has been described earlier that hydra undergoes morphallactic regeneration to restore its unique structure after amputation. Tentacles emerged within 30–36 h after amputation, and the head was fully regenerated within 48–72 h. This dynamic process of regeneration in hydra was altered on exposure to an environmental stress^38^. In our experiment hydra transversely bisected just below the head region and slightly above the gastric region were allowed to regenerate in the presence of sub-lethal concentrations of CuO NR for 72 h. Head regeneration of hydra exposed to 2.5 μM CuO NR was retarded mostly at 48 h post-amputation and 100% mortality was noticed after 72 h incubation whereas on exposure to a lower concentration of CuO NR tentacles emerged at 48 h of amputation and complete regeneration occurred within 72 h (Fig. 3E). These data clearly showed that CuO NR caused impairment of regeneration in a dose-dependent manner.

Epithelial cells play a significant role in regeneration and reproduction in hydra. The inhibition of regeneration was dose-dependent and it was caused due to inhibition of the proliferation of epithelial cells. To address if CuO NR exposure also influenced population growth rate in hydra, four polyps, each with a single bud, exposed to sub-lethal concentrations of CuO NR for 24 h, were fed and monitored continuously for 14 days. The growth rate constant *k* was calculated using the equation *ln* (*n*/*n*^0^) = *kt*. From Fig. 3F it is implicit that there was no significant difference in the *k* value between hydras treated sub-lethal doses of CuO NR and those not treated. These data clearly showed that short-time exposure of CuO NR did not impact the growth rate and reproductive capabilities of hydra to any great extent.

### Cell cycle analysis in hydra exposed to CuO NR

**Figure 4:**
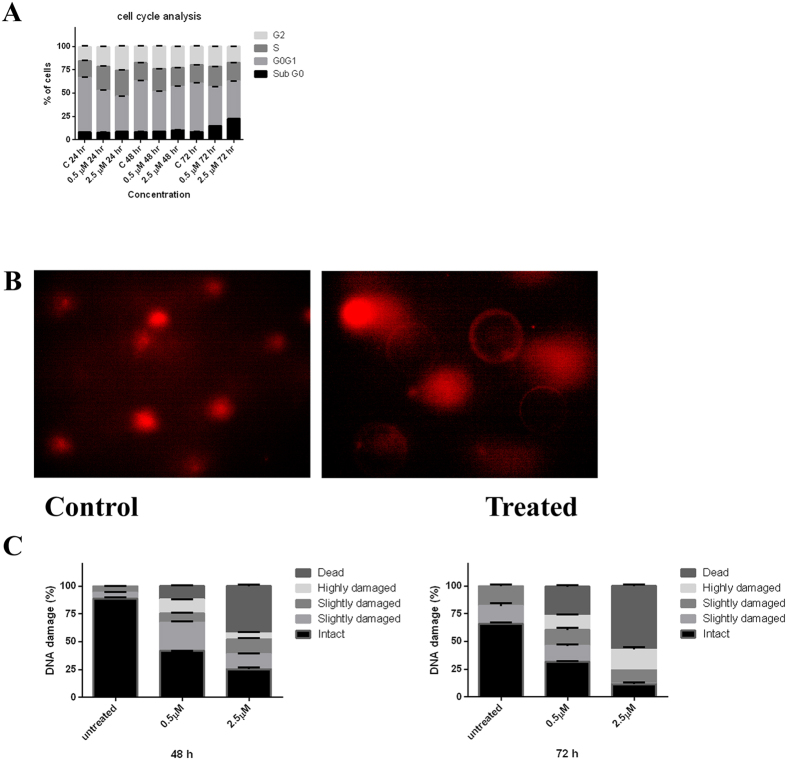
Cell cycle analysis and genotoxicity assessment. (**A**) Cell-cycle analysis of hydra exposed to various concentrations of CuO NR at different time points. The DNA content was determined using flow cytometry by staining with propidium iodide and the post-analysis was done with FlowJo software based on Watson (Pragmatic) model. Data are expressed as mean ± SE of three independent experiments. (**B**) Representative image of DNA damage in normal and CuO NR-exposed hydras as assessed by comet assay. (**C**) DNA damage is classified based on DNA in the tail. The stacked column (from bottom to top) corresponds to intact (0–20%), slightly damaged (20–40%), damaged (40–60%), highly damaged (60–80%), and dead (80–100%). Data are expressed as mean ± SE of three independent experiments.

Exposure of hydra to CuO NR resulted in accumulation of cells differentially at G_1_, S and G_2_ phases, depending on the dose as well as duration of exposure, and subsequent accumulation at sub-G_0_ phase to take to the apoptotic pathway. To investigate the influence of CuO NR on hydra cell cycle, cells of treated animals were subjected to flow cytometry and the results extracted were analyzed using FlowJo software. The data thus obtained revealed that at both the concentrations CuO NR treatment caused S-phase arrest during the first 24 h of exposure and the number of cells that underwent apoptosis was drastically increased on 48 and 72 h exposures (Fig. 4A). In the control hydras most of the cells were in G_1_-phase for all three time points, whereas in the treated hydras the cells exhibited concentration-dependent S- and G_0-_phase arrest. This pattern of cell cycle distribution suggested that CuO NR induced DNA damage and cell death in hydra in a concentration- and time-dependent manner.

### Genotoxicity assessment of hydra cells by comet assay

Genotoxicity or DNA strand break in hydra following exposure to CuO NR was assessed by subjecting the cells of treated animals to single cell gel electrophoresis followed by scoring the normal and comet-possessing cells and then making measurement of the size of the comet adopting CASP software. As shown in Fig. 4B DNA damage was produced at both the concentrations at 48 as well as 72 h time points. In the case of the latter the DNA damage was intense as revealed in the significant increase in tail length of the comets. Since the tail length and DNA in the tail are indicative of the extent of DNA damage, the percentage of DNA in the tail presented in Fig. 4C provides a quantitative measure of the damaged DNA.

### Induction of ROS generation in hydra exposed to CuO NR

**Figure 5:**
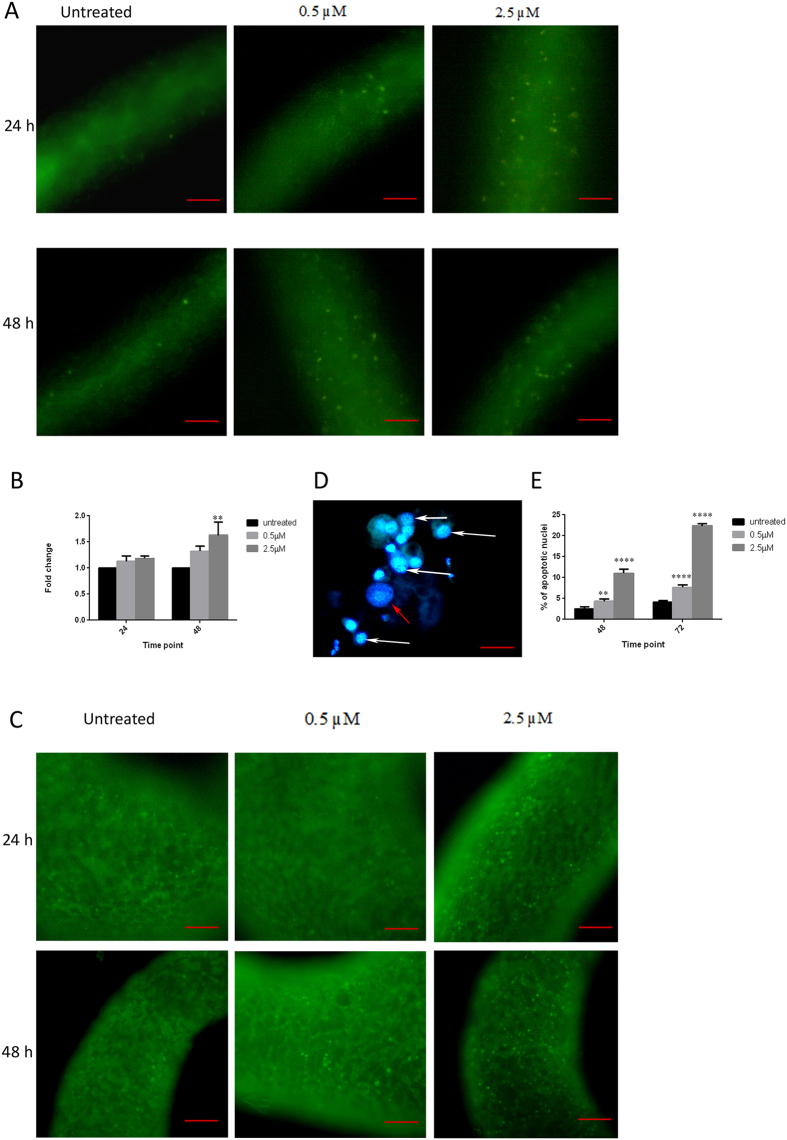
Fluorescent microscopic images of ROS and apoptosis in hydra following exposure to CuO NR. (**A**) Representative images of ROS generation in hydra following CuO NR exposure. Induction of ROS generation was revealed in the punctated green fluorescence. (**B**) Quantification of intracellular ROS generation in hydra following CuO NR exposure. ROS level is expressed as fold change in the fluorescence in comparison with untreated polyp. Significance between the control and the treatment groups were performed by adopting two way ANOVA with Dunnet’s multiple comparison test (**p < 0.01). (**C**) Representative fluorescent microscopic images of AO stained hydra following CuO NR exposure. Cells underwent apoptosis was revealed in the punctated green fluorescence. (**D**) Representative fluorescent microscopic images of DAPI-stained individual cells of hydra following CuO NR exposure White arrow indicates apoptotic cell in CuO NR exposed polyp; red arrow indicates normal cell in control polyp. (**E**) Quantitative analysis of DAPI-stained apoptotic nuclei following exposure to varying concentrations of CuO NR incubated for 48 h and 72 h. Data represent mean ± SE of three independent experiments. Multiple comparison between the untreated and treatment groups were performed by adopting two way ANOVA with Dunnet’s test (**p < 0.01, ****p < 0.001). Scale bar (**A**,**C**), 50 μM; (**D**) 1 μM.

The ability of CuO NR to induce oxidative stress was detected using the non-fluorescent dye 2, 7-dichlorofluorescin diacetate (H_2_-DCFDA). Accumulation of ROS results in oxidation of H_2_-DCF resulting in highly fluorescent DCF, which was observed as the green fluorescent punctae in the polyps. Results in Fig. 5A clearly demonstrated the increase in fluorescent punctae, *visa vi* the ROS, in a concentration- and time- dependent manner in CuO NR treated hydras. The increase in ROS generation was further confirmed by quantification of the DCF fluorescence in cell lysate of hydras (Fig. 5B).

### Induction of apoptosis

The mechanism by which cells of CuO NR-exposed hydra underwent death was evaluated by staining the cells with acridine orange and DAPI. Acridine orange (AO) is a fluorescent dye that highlights cells undergoing apoptosis as well as epithelial cells which have phagocytosed apoptotic cells, each in a manner very specific. The results, fluorescent green punctae, clearly revealed apoptotic cell death in the treated hydra (Fig. 5C). DAPI is a nuclear stain that specifically binds with the nucleus of the cell. DAPI-stained cells revealed condensed chromatin and fragmented nuclei which are hallmarks of apoptosis (Fig. 5D). Quantitative analysis of apoptosis in dissociated cells of hydra revealed that CuO NR induced apoptosis in a concentration- and time-dependent manner. Hydra incubated with 2.5 μM CuO NR for 72 h exhibited 23% nuclear damage which was the highest incidence (Fig. 5E).

### Alterations produced by CuO NR in expression of pro- and anti-apoptotic, antioxidant and stress-responsive genes

**Figure 6:**
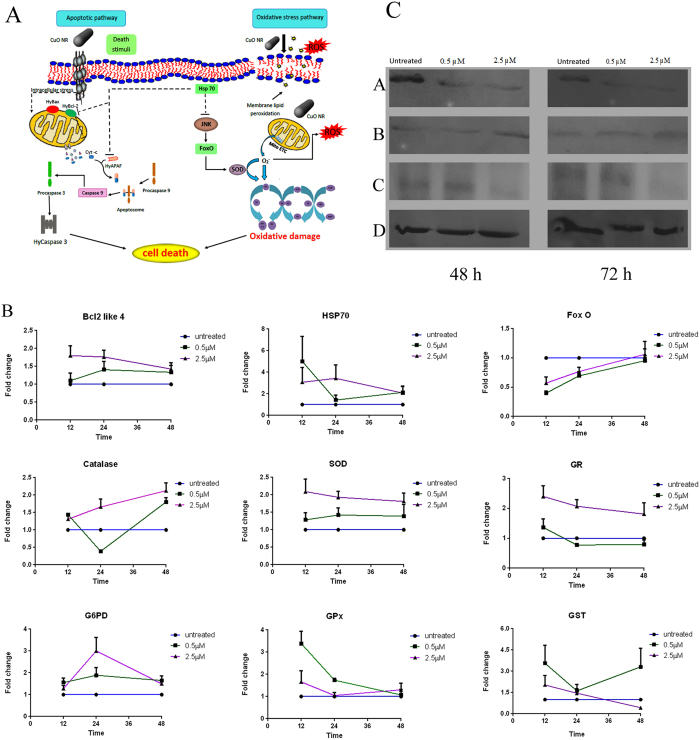
Schematic representation, gene expression and protein expression profiling in hydra exposed to CuO NR. (**A**) Schematic representation of molecular mechanism underlying toxicity induced by CuO NR. Left panel indicates the major proteins involved in apoptotic pathway and right panel indicates key components of oxidative stress pathway. Straight line indicates activation and dotted line indicates inhibition. (**B**) Gene expression profiling of selected genes of antioxidant defense system following CuO NR exposure using α-Tubulin as reference gene. Data represent mean ± SE of three technical replicates from three different biological samples. (**C**) Western blot analysis of selected proteins associated with apoptotic pathway. a) Βcl-2 b) Βax c) pro-caspase 3 d) β-actin.

It is generally known that nanomaterials induce oxidative stress in the biological systems. Figure 6A is the schematic representation of cellular response following CuO NR exposure. Release of reactive oxygen molecules modulates many signaling pathways leading to cell death. In order to validate if this is true in the present test system we investigated the changes in transcript levels of antioxidant genes (CAT, G6PD, GPx, GR, GST and SOD), anti-apoptotic gene (Bcl-2), apoptosis-regulatory gene (HSP70) and stress-responsive transcription factor FoxO. Figure 6B shows the data obtained at different time points, 12, 24 and 48 h, adopting qPCR using specific primers. Transcript levels of GPx gene were up-regulated at 12 h and decreased to normal level at 48 h in response to the lower concentration whereas overlapping expression was recorded at the higher concentration of CuO NR. In the case of G6PD, overlapping expression was observed at both the concentrations of CuO NR. The SOD transcript levels increased whereas those of GR decreased with increase of CuO NR in a concentration-dependent manner. Overlapping expression of catalase and GST was observed following exposure to the lower concentration; however, catalase was up-regulated at the higher concentration whereas GST was down-regulated in a time-dependent manner. Expression of anti-apoptotic gene Bcl-2-like 4 was found to be up-regulated at 12 h followed by down-regulation at 24 and 48 h for the higher concentration, whereas the expression was up-regulated at 24 h and down-regulated at 48 h following exposure to the lower concentration. Hsp70, a regulatory gene, showed overlapping expression at the lower concentration, but increased at the higher concentration during exposure for 12 h but decreased to normal at 48 h. This result indicated that at a higher concentration CuO NR induced apoptosis at nominal level by inhibiting the anti-apoptotic regulatory role played by Hsp70. Expression of stress-responsive transcription factor FoxO was down-regulated at 12 and 24 h at both the concentrations and at 48 h the expression was similar to control.

### Pro- and anti-apoptotic protein expression

To investigate the mechanism underlying cell death following CuO NR exposure in hydra, western blotting was performed. This approach revealed activation of apoptotic pathway in hydra via down-regulation of anti-apoptotic protein Bcl-2, up-regulation of pro-apoptotic protein Bax, and decrease of executioner pro-casapase 3 implying increased activation to caspase 3 (Fig. 6C).

## Discussion

The toxicity of nano CuO would differ in respect of its method of synthesis, physicochemical properties and the model in which it is tested^11^. Therefore, it is rather difficult to bring up a universal generalization of the toxic manifestations of nano CuO and the mechanisms of action. Hence, in the present study we tested the effect of CuO NR in a phylogenetically lower ranking invertebrate, *Hydra magnipapillata* 105. Unlike the prokaryotes and cell models, invertebrate organisms offer several advantages in the context of nanotoxicology such as the ability to absorb nanomaterials from the aquatic environment in several ways, i) direct ingestion or by ingestion of contaminated prey organisms, ii) water filtration, iii) inhalation, and iv) direct contact, and metabolize them^19^. But, then the different invertebrates differ in complexity and so the manifestations to toxic insult is also either different or differ in magnitudes. Here, hydra offers unique advantages in that it is a simple aquatic organism that can be easily cultured in the laboratory. It can access the test material present in the medium from both outside (through the ectoderm) and inside (through the gastrodermis which lines the gastrovascular cavity which is ensured with a continuous flow of water current). Being an organism it provides for cell-cell interaction and so the manifestation, though technically at the level of cell, is revealed in the whole body. Thus, hydra provides the special advantage of a very simple and efficient *in vivo* test system, also providing scope for analysis at the level of individual cells^33^. The data presented here demonstrate the effect of CuO NR at whole animal, cellular and molecular levels and facilitate understanding of the mechanisms that underlie the toxicity of CuO NR in this simple organism. Owing to the continuous multiplication of cells in hydra, chronic exposure allows all the renewing cells to be exposed to the CuO NR thus facilitating understanding of the long-term effects of the toxicant.

Severe morphological changes and lethality that were observed in hydra following exposure to increasing concentrations of CuO NR facilitated us to determine its median lethal concentrations (LC_50_) and its comparison with CuSO_4_. Interestingly, lower toxicity was observed in hydra exposed to CuO NR than to the same mass concentration of CuSO_4_ (Supplementary information Fig. S2). These results are in good agreement with the previous findings in *Daphnia similis*^39^, *Daphnia magna, Thamnocephalus platyurus*, *Tetrahymena thermophila*^40^, and *Lemna gabba*^41^, where nano CuO produced lower toxicity than the bulk CuSO_4_. Lower toxicity of CuO NR may be due to the solubilization i.e., instability of the nanorods in the medium.

The available literature suggested that release of Cu^2+^ from the core shell of CuO nanostructures induces toxicity when it exceeds the physiological tolerance limit^42^,^43^. Therefore, to check if the release of Cu^2+^ from CuO NR has any adverse effect on hydra, the intracellular concentration of Cu^2+^ was determined after exposing live polyps to sub-lethal concentrations of CuO NR. The results indicated that internalization of Cu^2+^ was higher when the concentration in the medium was higher and vice versa. The Cu^2+^ content of hydra (calculated from 150 treated animals) after incubation for 24 h was not commensurate with the concentration in the medium. The poor accumulation of Cu^2+^ in CuO NR-treated hydra and the lesser toxicity may be attributed to the adherence of the NR, in view of their large surface area, to the organism’s outer shell such that the entry of the material inside the organism is hindered, particularly by the cuticle which would act as a barrier and permit penetration of foreign particles only selectively as suggested by Ambrosone *et al*.^37^.

The adverse effects imposed by sub-lethal concentrations of CuO NR on the specific cellular arrangement and feeding behavior were determined following disruption of battery cell complexes and impairment of feeding behavior. The results suggested that CuO NR depleted the battery cell population and produced no impact on hydra’s response to glutathione as observed in tentacle writhing. Glutathione, when added to the medium, elicits the response through initiating a signaling mechanism. This does not seem to be affected by exposure to CuO NR since there is no scope for the tentacles/BCCs handling an object. On the other hand BCCs are concerned with capturing the prey, and the tentacles by and large physically handle the nauplii. Since CuO NRs affected the BCCs the latter did not respond to the prey properly and also the tentacle writhing was impaired.

In order to determine the long term effect of CuO NR on the biologically important processes such as growth and regeneration, hydra was exposed to two different sub-lethal concentrations of the NR. It was seen that CuO NR has little impact on the growth rate at both the concentrations, which corroborates the lack of response in terms of feeding, as discussed *vide supra*, but it is interesting that regeneration was inhibited by exposure to the higher concentration. The latter may be due to permeation of the nanomaterial into the cells to interact with the DNA and induce DNA damage as in the context of inhibition of caudal fin regeneration in zebra fish^44^. But in our study the internalization of Cu^2+^ was far less than the estimated LC_50_ that inhibits regeneration. This may be due to an inherent property of the nanorods, which needs further investigation. The impairment of regeneration at only the higher concentration may be due to the threshold toxicity value of CuO NR. Cell cycle analysis further confirmed S-phase arrest during 24 h treatment and revealed induction of DNA damage following CuO NR exposure. Cell cycle analysis after 48 h and 72 h exposures showed accumulation of most of the cells in Sub-G0 phase with corresponding decrease of cells in G0/G1 phase. This pattern of accumulation indicates apoptotic cell death^45^. Thus, the cell cycle analysis provided evidence for induction of irreversible DNA damage followed by apoptotic cell death as caused by exposure to CuO NR.

Induction of oxidative stress has been proposed to be one of the mechanisms underlying cell death induced by most of the metal oxide nanoparticles^46^. Oxidative stress ensues as a consequence of imbalance between generation of ROS and the counteracting antioxidants in the biological system. In this study we observed that CuO NR, even at a very low concentration, induced ROS generation. Thus, we hypothesized that ROS generation in the present context may be the ultimate cause of cell death in hydra, acting through DNA damage and failure of DNA repair mechanism. Induction of ROS generation in hydra can be possibly explained from two perspectives. 1) Nanomaterials that are engulfed by the cells are delivered to the lysosomes. Since the lysosomes are acidic in nature (pH 4.5) nano CuO dissolves in the lysosomes and thus Cu^2+^ is released^3^. The release of Cu^2+^ induces ROS generation (Fenton reaction) which ultimately leads to DNA damage and initiation of caspase cascade. In the present study we measured Cu^2+^ in the treated animal which came out to be too low to cause mortality (Fig. 2C). 2) Induction of ROS generation by nano CuO is due to the nanoparticulate nature itself rather than release of Cu^2+^ from nano CuO^14^. Earlier to this, Bandara *et al*.^47^ demonstrated that nano CuO is capable of generating H_2_O_2_ using O_2_ in the presence of light. In the present case we inferred that induction of ROS generation by CuO NR is due to its large surface area and the incubation of the experimental set up in bright light rather than the dissociation of CuO in the lysosomes to release Cu^2+^.

Finally, the antioxidant defense mechanism that would deal with the excess free radicals in terms of alterations of transcription of genes that encode antioxidant defense molecules was assessed. Cellular responses against free radicals commence at transcriptional level and, hence, activation of genes that encode molecules concerned with antioxidant system were monitored and measured at different time points. Gene profiling by qRT-PCR revealed clear concentration- and time-dependent alterations in expression of CAT, G6PD, GPx, GR, GST, SOD, FoxO and Hsp70 genes, which showed hydra’s controlled response in maintenance of homeostatic condition of the body. At the higher concentration the stress-responsive antioxidant genes CAT, G6PD, GPx, GR and SOD, and stress-responsive regulatory genes Hsp70 exhibited increased expression at all time points; however, the transcript level of GST was found to decrease at 48 h. GST plays a significant role in counteracting the excess ROS through its peroxidase activity. However, reduced expression of GST resulted in decreased synthesis of GST protein which is not sufficient to act later on to detoxify the free radicals that were induced^23^. These findings suggest that CuO NR not only induces oxidative stress but also possesses the ability to inhibit antioxidant defense system. At the lower concentration the transcripts levels of genes CAT, G6PD, GST, and SOD exhibited overlapping expression and the increased expression of the GPx and GR genes, observed at 12 h, decreased to normal level at 24 and 48 h which suggest that these genes do not play any significant role when the exposure is to a concentration lesser than the threshold. Overall, these findings suggest that exposure of hydra to a higher concentration of CuO NR increased the susceptibility to cell death by way of increased ROS generation and failure of the antioxidant defense system, whereas following exposure to a lower concentration of CuO NR hydra produced counteracting antioxidant defense system to neutralize the enhanced oxidative stress and activated the adaptive mechanisms by up-regulating the stress-responsive gene Hsp70. Failure of defense system to neutralize excess ROS generation inflicts deadly cellular effects such as lipid peroxidation^42^, protein oxidation^48^, DNA damage and apoptosis^49^.

Activation of stress signaling pathways induces DNA damage and reduces cell proliferation rate, eventually leading to activation of apoptotic cell death. The activation of apoptosis pathway following CuO NR exposure was qualitatively determined by DAPI staining. Increased number of apoptotic cells was observed on exposure to the higher concentration CuO NR from 48 h onwards. Apoptosis is generally induced via death receptor-mediated extrinsic pathway and/or mitochondria-mediated intrinsic pathway. Induction of either or both the pathways would culminate in activation of pro-caspase-3 into caspase-3, resulting in cleavage of DNA, fragmentation of chromatin and formation of apoptotic bodies^50^. In our study CuO NR exposure decreased the expression of anti-apoptotic protein Bcl-2, decreased the level of pro-caspase-3 *visa vi* increased the activation of pro-caspase 3 into active caspase 3, and increased the expression of pro-apoptotic protein Bax. These results substantiate the occurrence of apoptosis in hydra following exposure to CuO NR along the mitochondria-mediated intrinsic pathway.

## Conclusion

A comprehensive study to decipher the mechanisms underlying the toxicity induced by CuO NR in *Hydra magnipapillata* 105, a simple invertebrate organism, was undertaken. The study clearly shows that CuO NR produced adverse effects in hydra in terms of morphology, feeding, growth and regeneration. Further, *in vivo* experiments followed by *in vitro* analysis provided tangible information about lethal effects at cellular level, including induction of oxidative stress, S-phase cell cycle arrest and toxicological implications at cellular and molecular levels. Hydra exhibited clear sign of toxicity to CuO NR in a concentration- and time-dependent manner leading to adverse effect on regeneration and induction of genotoxicity. Exhibition of molecular responses even in the absence of evident morphological alterations and high sensitivity to inorganic metal oxide nanomaterials render hydra an amenable and convenient organism for fresh-water ecotoxicological risk assessment of nanoparticles.

## Materials and Methods

### Synthesis of CuO NR

All reagents used for CuO NR synthesis were analytical grade and used without further purification. Highly dispersed CuO NR was synthesized according to Zhu *et al*.^51^. One mL of glacial acetic acid was added to 300 mL of 0.02 M aqueous copper acetate solution in a round-bottomed flask connected to a refluxing device. The mixture was heated at 60 °C with vigorous stirring, and then 0.8 g of sodium hydroxide pellet was quickly added until the pH of the mixture turned 6–7. The black precipitate that formed was cooled to room temperature and centrifuged to collect the precipitate. The precipitate was washed once with distilled water and thrice with absolute ethanol, and dried in air at room temperature.

### Physicochemical characterization of CuO NR

Transmission electron microscopy (TEM, Philips CM20), at an accelerating voltage of 200 kV, was employed to study the morphology of the nano-rod thus synthesized. Ten nanorods at random were subjected to measurement of length and breadth from which values the respective means and standard deviations were calculated using GraphPad Prism 6.0 software. X-Ray Diffraction (XRD) measurements were performed using PANalytical X PERT PRO PANalytical X-ray diffractometer with CuKα radiation (40 kV, 30 mA). The particle was sonicated in a bath sonicator for 20 min (53 kHz) at room temperature to determine the zeta potential in water using dynamic light scattering (DLS) (Nano-zetasizer-HT, Malvern Instrument, UK). GeneQuant 1300 UV-Vis spectrophotometer was used to record the absorption spectra.

### Culture of *Hydra magnipapillata* 105

The polyps were cultured in hydra medium (1 mM CaCl_2,_ 1 mM NaCl, 0.1 mM MgSO_4_, 0.1 mM KCl, and 1 mM Tris-Cl pH 7.8) at 18 °C under 12 h dark-light cycle^52^. The animals were maintained at 18 ± 1 °C and fed once every two days with freshly hatched *Artemia* nauplii.

### Morphological analysis

#### Acute toxicity testing of CuO NR in hydra

Semi-static acute toxicity testing in hydra was conducted according to Troitter *et al*.^53^. Briefly, 25 live polyps without bud were placed in petridishes containing 25 mL of hydra medium (cf. *vide supra*) and cultured at 18 ± 1 °C under 12 h dark-light cycle. The polyps were continuously exposed to varying concentrations of copper oxide nanorods (CuO NR) ranging from 0.5 μM to 3.5 μM for 72 h and the morphological changes were recorded at 24 h by observation at x20 magnification in a stereo-zoom dissecting microscope (Carl Zeiss, Jena, Germany). A score of 10 was assigned to highly intact and healthy polyps and 0 to animals that had undergone disintegration, with scores 9-1 indicating pathologies in that order adopting Wilby’s^34^ scale of toxicity testing (Supplementary Table S2). The median score was analyzed to determine median lethal concentration for each time point.

#### Tentacle structure and disruption of battery cell complex (BCCs)

Disruption of battery cells in tentacles was observed according to Ambrosone *et al*.^37^ in a bright field microscope adopting toluidine blue staining. Briefly, the animals after the treatment were relaxed in 2% urethane for 1 min and then fixed with absolute ethanol for 5 min. The animals were repeatedly washed with distilled water and stained with 0.05% toluidine blue prepared in 10 mM Tris-Cl (pH 7.5). The excess dye was removed by washing the animals in distilled water and the animals were dehydrated in grades of ethanol. The animals were cleared and mounted in DPX mountant. The stained tentacles were examined under bright field and phase contrast illuminations in a research microscope (Axioskop 2 plus, Carl Zeiss, Jena, Germany) equipped with Axiocam ERc5 s camera.

### Feeding assay

Feeding assay was performed according to Ambrosone *et al*.^37^. CuO NR-exposed and -unexposed polyps were challenged with freshly hatched *Artemia* nauplii or 10 mM reduced glutathione (GSH). Hydra’s ability to trap the prey and respond to glutathione molecule was observed in a stereo-zoom dissecting microscope (Carl Zeiss, Jena, Germany) equipped with Axiocam ERc5 s camera.

### Determination of copper content by elemental analysis

The intracellular copper content was determined by incubating 150 polyps in 0.5 μM and 2.5 μM CuO NR in 25 mL of hydra medium for 24 h. After incubation hydras were digested in aqua regia and the resulting filtrate was subjected to determination of copper concentration by inductively coupled plasma atomic emission spectroscopy (ICP-AES). Experiments were conducted in triplicate and the mean value is presented as copper content per hydra.

### Hydra growth rate and regeneration

Growth rate determination in the presence of CuO NR was performed according to Ambrosone *et al*.^18^ Untreated animals and animals exposed to sub-lethal concentrations of CuO NR for 24 h (four hydras, each with one bud) were rinsed in hydra medium and subsequently placed in multi-well plates (1 hydra/well). Both treated and untreated polyps were fed once every day for 14 days. The growth rate constant (k) was calculated using the formula ln (n/n0)/t = k where n is the number of animals at time t (day of the experiment) and n0 the number of animal at t0 i.e., initial day of the experiment. T2, the doubling time of the population was determined by linear regression.

For regeneration experiments, groups of 15 polyps were amputated in the upper gastric region and allowed to regenerate the missing part in the presence of sub-lethal concentrations of CuO NR. Polyps that were undergoing regeneration were examined in a stereo-zoom dissecting microscope and scored 0 (no regeneration), 1 (emergence of tentacles) and 2 (fully regenerated), according to Ambrosone *et al*.^18^.

### Cell cycle analysis by flow cytometry

Cell cycle analysis was performed according to Bugzariu^54^ with slight modifications. Briefly, after the treatment hydra were collected in a centrifuge tube, washed and digested in 250 μl of trypsin-EDTA for 5–7 min at 37 °C to dissociate the cells. The centrifuge tubes were kept on ice and trypsin was inactivated by addition of 100 μL of fetal calf serum (FCS) to the cell suspension. 5*10^5 ^cells/mL were fixed in 4% paraformaldehyde and permeabilized in 70% ethanol. Cells were then stained with 500 μL of hypertonic NP-40 buffer (Propidium Iodide (PI) 40 μg/mL, RNase A 200 μg/mL, 0.5% NP-40 in PBS), for 30 min. The fluorescence emission was measured on a FACS Calibur II System (Becton–Dickinson) using an argon-ion laser at 488 nm, together with the forward scattered (FSC) and side scattered (SSC) parameters. The emitted PI fluorescence was collected by a 585 nm bandpass filter in the FL2 channel. Typically 10,000 events per sample were collected using the Cell Quest software, and analyzed with FlowJo software (TreeStar Inc) after excluding debris, clumps (FSC versus SSC) and doublets (signal area FL2-A versus signal width FL2-W). All experiments were performed in triplicate to facilitate statistical analysis.

### Determination of ROS

#### H_2_-DCFDA staining

ROS generation at whole animal level was observed using the non-fluorescent dye H_2_-DCFDA, according to Jantzen *et al*.^55^. Briefly, polyps exposed to CuO NR were incubated with 10 μM H_2_-DCFDA dye for 1 h in dark. The polyps were thoroughly washed in hydra medium, mounted on 2% urethane and immediately observed in the fluorescent microscope (Axioskop 2 plus, Carl Zeiss, Jena, Germany).

#### Quantification of intracellular ROS level

Intracellular level of ROS was determined according to Keston *et al*.^56^. Cell lysate was prepared in PBS by homogenizing hydra using a micropipette. The supernatant was collected by centrifuging the cell lysate at 15,000 × g at 4 °C for 15 min. The protein concentration was determined using Bradford reagent with bovine serum albumin (BSA) as the standard^57^. Intracellular ROS level was determined by incubating the cell lysate representing each treatment with 10 μM H_2_-DCFDA dye for 20 min in dark. The ROS release was monitored by the conversion of non-fluorescent H_2_-DCFDA dye into highly fluorescent 2′, 7′-dichlorofluorescein (DCF) using a fluorometer (Perkin Elmer, USA).

### RNA isolation and Real Time PCR (qPCR)

Total RNA from untreated and treated animals was isolated using the TRIzol Reagent (Invitrogen; USA) and quantified using a NanoDrop spectrophotometer (BioDrop Duo, UK). First strand cDNA was synthesized with 1 μg of RNA and oligo dTs to a final volume of 20 μL according to the manufacturer’s instruction (Thermo Scientific, USA). qPCR was performed as 10 μL reaction with a final concentration of 1X SYBR Green (Roche), 0.5 μL of each primer, and the cDNA that was synthesized in the earlier step. The reactions were carried out using single step real time PCR machine (Roche, USA) under the following conditions: initial denaturation at 95 °C for 5 min, followed by 30 cycles of 3 step amplification at 95 °C for 10 s, 48/52 °C for 30 s and 72 °C for 10 s (annealing temperatures of individual primers are listed in the Supplementary Table S3). Primers for the genes Superoxide dismutase (HySOD), Forkhead boxO (FoxO), Heat shock protein (Hsp70), Bcl2-like 4, Catalase (CAT), Glucose-6-phosphate dehydrogenase (G6PD), Glutathione peroxidase (GPx), Glutathione reductase (GR), and Glutathione S-transferase (GST) were selected from the earlier reports of Woo *et al*.^32^ and Ambrosone *et al*.^18^ (the sequences of individual primers are listed in the Supplementary Table S4). In addition, melting curves (95 °C for 10 s; 65 °C for 60 s and 97 °C for 1 s continuous) were generated to check any spurious amplification products. α-Tubulin 1 served as the internal control to normalize the RNA level. Three technical repeats and experimental replicates were performed for each gene.

### Single cell gel electrophoresis (Comet assay)

Genotoxicity assessment in hydra was conducted according to Kovacevic *et al*.^58^ with slight modification. Briefly, after the treatment hydras were collected in a centrifuge tube and washed with hydra medium. The animals were digested in 250 μL of trypsin-EDTA for 5–7 min at 37 °C. After placing the tube on ice, trypsin was inactivated by addition of 100 μL of fetal calf serum (FCS) to the cell suspension. This cell suspension, mixed with low melting agarose in PBS, was placed on microscopic slides and covered with normal melting agarose. Slides were immersed in pre-chilled lysis buffer (2.5 M NaCl, 100 mM Na_2_-EDTA, 10 mM Tris, 0.2 mM NaOH [pH 10], 10% DMSO and Triton X-100) and incubated overnight at 4 °C. Alkaline denaturation and gel electrophoresis were performed in cold condition under dim light in freshly prepared electrophoresis buffer (300 mM NaOH, 1 mM Na_2_-EDTA, [pH > 13]) for 20 min at 20 V. Then the slides were washed with neutralization buffer (0.4 M Tris, pH 7.5) and observed in the fluorescent microscope. Three hundred cells from each treatment group were captured and analyzed using CASP software. Tail intensity and tail length were measured to assess the DNA damage. Experiments were performed in triplicate and the mean value was calculated to evaluate the degree of DNA damage representing the fraction of total DNA in the tail.

### Detection of apoptosis

#### Acridine orange staining

Apoptotic cells in whole animal were observed according to Cikala *et al*.^59^ using acridine orange dye. Briefly, polyps exposed to CuO NR were incubated with 3.3 μM of acridine orange for 15 min in dark. Polyps were washed adequately with hydra medium, relaxed in 2% urethane and observed in the fluorescent microscope.

#### DAPI staining

Apoptosis at cellular level was determined by staining the untreated and treated cells with 4′-6-Diamidino-2-phenylindole (DAPI) dye according to Cikala *et al*.^59^. Briefly, fixed single cell suspensions of macerated hydras (treated or untreated) were spread separately on gelatin-coated microscopic slides. After adequate wash in PBS the cells were stained with DAPI for 2 min and washed in PBS. Cells were examined in a phase contrast fluorescent microscope (Axioskop 2 plus, Carl Zeiss, Jena, Germany). 300 cells were examined for each treatment and the percentage of apoptotic cells was calculated.

### SDS-PAGE and western blotting

Total protein was extracted from treated and untreated polyps using Dignam buffer (Hepes 10 mM, MgCl_2_ 1.5 mM, KCl 10 mM, Cocktail Protease inhibitor from Roche and NP 40 0.1%)^60^. Protein concentration was determined adopting Bradford method^2^ with bovine serum albumin (BSA) as the standard. 10% SDS-PAGE was performed for protein separation, and western blotting was performed with Bcl-2, Bax and pro-casapase 3 antibodies, at 1:1000 dilution.

### Statistical analysis

The data were subjected to statistical analysis using Graph Pad Prism-6.0 and the results are expressed as mean ± SE of three independent experiments. Data in respect of acute toxicity testing, disruption of battery cell complexes, ROS generation and apoptosis were subjected to two way ANOVA with Dunnet’s multiple comparison test. For regeneration assay chi-square test was adopted.

## Additional Information

**How to cite this article**: Murugadas, A. *et al*. Hydra as a model organism to decipher the toxic effects of copper oxide nanorod: Eco-toxicogenomics approach. *Sci. Rep.* **6**, 29663; doi: 10.1038/srep29663 (2016).

## Supplementary Material

Supplementary Video S1

Supplementary Video S2

Supplementary Video S3

Supplementary Video S4

Supplementary Information

## Figures and Tables

**Table 1:** Median lethal concentration (LC_50_) was calculated by adopting probit analysis for each time point. Scores ≥6 were reversible and sub-lethal, while scores ≤5 were considered irreversible and lethal.

Time (h)	95% confidence limits for concentrations (μg/L)		
	Estimate	Lower bound	Upper bound
24	437.89	429.23	441.12
48	216.16	209.446	222.90
72	157.68	152.26	162.98
