# Supplementary material for: Hydra as a model organism to decipher the toxic effects of copper oxide nanorod: Eco-toxicogenomics approach
Source: Sci Rep. 2016 Jul 15;6:29663. doi: 10.1038/srep29663 (PMC4945869; doi:10.1038/srep29663)
Supplement: Supplementary Information [file srep29663-s5.pdf]

Hydra as a model organism to decipher the toxic effects of copper oxide nanorod: Eco-toxicogenomics approach

Murugadas Anbazhagan [1, 2], Zeeshan Mohammed [1, 2], Thamaraiselvi Kaliannan [1], Ghaskadbi Surendra [3], Akbarsha Mohammad Abdulkader[* 2, 4]

[1] Department of Environmental Biotechnology, Bharathidasan University, Tiruchirappalli 620024, India

[2] Mahatma Gandhi - Doerenkamp Center for Alternatives to Use of Animals in Life Science Education, Bharathidasan University, Tiruchirappalli 620024, India

[3] Developmental Biology Group, MACS-Agharkar Research Institute, Pune 411004, India

[4] Department of Food Science and Nutrition, College of Food and Agriculture, King Saud University, Riyadh 11451, Kingdom of Saudi Arabia

[*]Corresponding author

Dr. M. A. Akbarsha

Director & Chair

Mahatma Gandhi-Doerenkamp Center, Bharathidasan University,

Tiruchirappalli – 620024, India,

Tel: +91-431-2407117, Mobile: +91-9790995854

mgdcaua@yahoo.in, akbarbdu@gmail.com

Video legends (Main article)

M1. Feeding behavior of hydra

M2. Hydra’s response to GSH (positive control)

M3. Alterations in feeding behavior of hydra exposed to 2.5 µM CuO NR for 24 h

M4. Hydra’s response to GSH after exposure to 2.5 µM CuO NR for 24 h

Supplementary information

Legends

Figure. S1. Median score of hydra exposed to same mass concentration of $CuSO_4$ compared with CuO NR.

Table S1. Median score of hydra exposed to CuO NR. Significance between the control and the treatment groups were performed by adopting two way ANOVA with Dunnet’s multiple comparison test (****$p < 0.001$, **$p<0.05$).

Table S2. Wilby’s scale to measure toxicity end points.

Table S3. Annealing temperature of primers employed in the qRT-PCR analysis. The primers were adopted from Woo et al. (2012) and Ambrosone et al. (2012).

Table S4. List of forward and reverse primers employed in the qRT-PCR analysis.

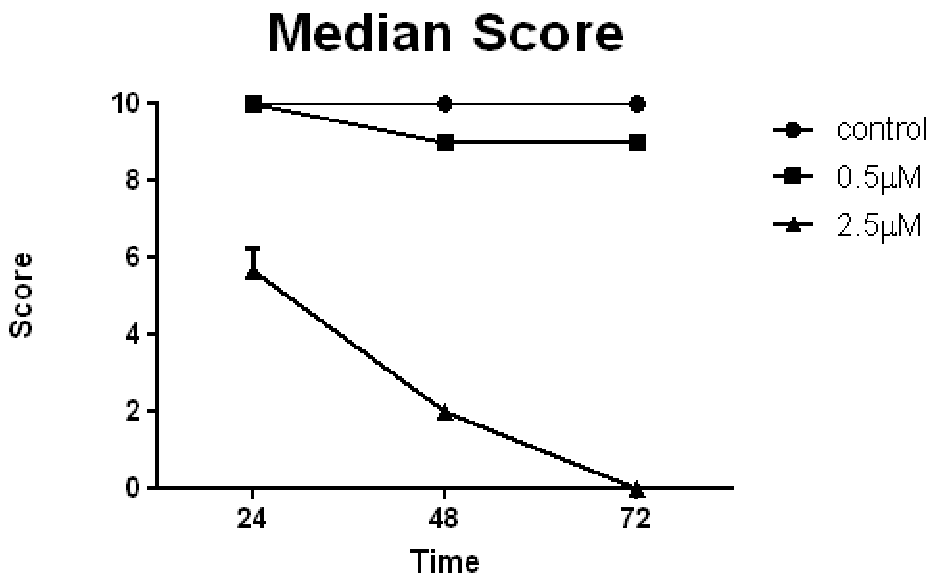

Figure. S1. Median score of hydra exposed to same mass concentration of $CuSO_4$ compared with CuO NR.

TABLES

**Concentration (μM)**	**Exposure time (hr)**		
	**24**	**48**	**72**
Untreated	10	10	10
0.5	10	10	10
1	10	10	10
1.5	10	9**	7****
2	10	7****	5****
2.5	10	6****	4****
3	10	5****	4****
3.5	10	4****	4****

Table S1. Median score of hydra exposed to CuO NR

Key for assessing progressive toxic effects in hydra polyps, Wilby’s scale (1988)	
Score	Morphology of polyp
10	Extended tentacles; body reactive
9	Partially contracted; slow reactions
8	Clubbed tentacles; body slightly contracted
7	Shortened tentacles; body slightly contracted
6	Tentacles and body shortened
5	Totally contracted; tentacles visible
4	Totally contracted; no visible tentacles
3	Expanded; tentacles visible
2	Expanded; no visible tentacles
1	Dead but intact
0	Disintegrated

Table S2. Wilby’s scale to measure toxicity end points

**Primer**	**Annealing temperature**
FoxO	48 ^{0}C
Hsp70.	52 ^{0}C
Bcl-2 like 4	52 ^{0}C
Catalase (CAT)	48 ^{0}C
G6PD	48 ^{0}C
GPx	52 ^{0}C
GR	48 ^{0}C
GST	52 ^{0}C
SOD	52 ^{0}C
TUB-α1	Both 48 ^{0}C and 52 ^{0}C

Table S3. Annealing temperature of primers employed in the qRT-PCR analysis. The primer designs were adopted from Woo et al., (2012) and Ambrosone et al., (2012)

FoxO	XP_002167754	GGATTTCCGATGCAAG TACG	TAAGTCTGCTTGGC GAACG	109
Hsp70.1	XP_002159813	CGACGTATTCAGACA ATCAACC	CAATTTGAGGAAC ACCTCTTGG	136
Bcl-2 like 4	XP_002167578	AACAAGGTGGATGGG ATGG	ATAAGTAATGCGC CCACACC	147
Catalase (CAT)	CN631284	GCTCCAAACTACTTCC CTAACAG	GCTCATCTATCGCT TCATTT	298
G6PD	DT614664	GCATTGCCACCATCTG TATTCA	GCAAACCTTAGCA CCATTAT	230
GPx	DQ286040	TCGATATCTGGAACCA ATGACAAA	CGAGGCGCCCACT ATGACTT	209
GR	XM_00215997 9	GAGGAGCGTATTTTGG GTAT	GTTTAACCTCAGCA ACCAGT	209
GST	XM_00215396 8	CGAGGCAGCTAAGTT AAAGT	ACTTAAGGTAATG GGGGATG	246
SOD	XM_00215747 1	TCAGTTTGGGGATTAT TCAGGTG	TCCAGCATTTCCGG TAGTTTTG	280
TUB-α1	CV660826	TTGATGAAATACGCAC AGGAACA	CCACCAAAGGAAT GAAAAAT	205

Table S4. List of forward and reverse primers employed in the qRT-PCR analysis.
